# Preliminary Study of the Mural Paintings of Sotterra Church in Paola (Cosenza, Italy)

**DOI:** 10.3390/ma15093411

**Published:** 2022-05-09

**Authors:** Michela Ricca, Maria Francesca Alberghina, Negin Derakhshan Houreh, Aybuke Sultan Koca, Salvatore Schiavone, Mauro Francesco La Russa, Luciana Randazzo, Silvestro Antonio Ruffolo

**Affiliations:** 1 Department of Biology, Ecology and Earth Sciences (DiBEST), University of Calabria, Arcavacata di Rende, 87036 Cosenza, CS, Italy; michela.ricca@unical.it (M.R.); francesca.alberghina@gmail.com (M.F.A.); silvestro.ruffolo@unical.it (S.A.R.); 2 S.T.Art-Test, 93015 Niscemi, CL, Italy; info@start-test.it; 3 Department of Conservation & Restoration of Cultural Properties, Faculty of Fine Arts, Ankara Hacı Bayram Veli University, Gölbaşı, 06570 Ankara, Turkey; hourehderakhshan.negin66@gmail.com (N.D.H.); aybukesultan@gmail.com (A.S.K.); 4 Department of Earth and Sea Sciences, University of Palermo, 90123 Palermo, PA, Italy; luciana.randazzo@unipa.it

**Keywords:** Sotterra church, mural paintings, frescoes, non-destructive analyses, pigments, infrared imaging, stratigraphy

## Abstract

A multi-analytical approach was employed to study wall paintings located in the *Sotterra* church at Paola, in the province of Cosenza, Italy. The site is an underground church (hence the name of Sotterra, which means “under the earth”) rediscovered in the second half of the 19th century, during the building works of the *Madonna del Carmine* church on the same area. This underground church preserves valuable mural paintings having different styles. The construction’s dating and overlapped modifications made until the site was abandoned is also debated. A wall painting, depicting “The Virgin” as part of the “Annunciation and the Archangel Gabriel” present on the opposite side of the apse, was selected and investigated using both in situ and laboratory-based analysis. Preliminarily, the non-destructive investigations involved several analytical techniques (IR imaging, UV-Induced Visible Fluorescence, and X-ray Fluorescence analyses) that provided mapping and characterization of pictorial layers and first data about deterioration phenomena. On the basis of this information, a more in-depth study was conducted on micro-fragments aimed at characterizing the stratigraphy and to identify the artist’s technique. Cross-sections were analysed using polarized optical microscopy and electron scanning microscopy coupled with energy-dispersive X-ray spectroscopy to obtain morphological and chemical information on the selected pictorial micro-fragments of the wall painting. The results allowed to characterize the pigments and provide better readability of the whole figure, revealing details that are not visible to the naked eye, important for future historical-artistic and conservative studies. The results represent the first step of a systematic archaeometric research aimed at supporting the ongoing historical-stylistic studies to distinguish the different building phases hypothesized for this religious site which remained buried for three centuries.

## 1. Introduction and Historical Background

The church of Sotterra, located in Paola in the province of Cosenza, near the Gaudimare district, is probably of Byzantine origin (between the 9th and 10th centuries) [1] but underwent significant changes with the advent of the Normans in Calabria beyond the 14th century, probably by Benedictine monks, as evidenced by the coexistence of wall paintings referable to these two periods.

The church, according to some historians, was built in the context of a hermit site [2,3], of which it was probably the fulcrum, in an area on the geographical border between the domains of Byzantium and the Longobard possessions. On the other hand, the region, between the 7th and the 9th centuries, was intensely affected by numerous waves of immigration of Eastern monks, who fled iconoclastic persecution and the Arab invasions of Egypt and Syria and, subsequently, of Sicily. Then, in accordance with the most sustained historical stylistic hypothesis, the most probable dating is placed by scholars between the 9th and 10th centuries [1] during the restoration of the dominion of Byzantium over the whole of Calabria and the Arab conquest of Sicily, which favoured the settlement on the Calabrian Tyrrhenian coasts of many Italian-Greek Byzantine monks. Its structural and stylistic stratifications make the underground church of Sotterra a cultural site of great interest representing one of the most important examples of Byzantine and medieval religious art in Calabria.

As the name suggests, the church is currently located below ground level and is a hypogeum site. Its original nature was different and was probably gradually covered by deposit material due to landslides and/or floods. The memory of this site, in fact, was probably completely lost in the 16th century and only in 1874, during the construction of the overlying church of the *Madonna del Carmine* was rediscovered through a hole in the open ground at the height of the presbytery vault [4,5]. The first systematic exploration of the Sotterra church was carried out by Claudio Ricca of the Superintendency of Bruzio and Lucania in 1925 [4].

**Figure 1:**
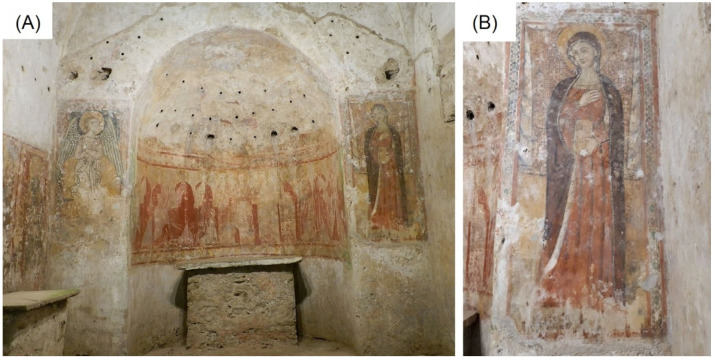
(**A**) Overview of the cycle of frescoes in the church of Sotterra with evidence, from left to right, of the angel of the Annunciation, Christ in glory with apostles and the Virgin. (**B**) Detail of the Virgin as part of the mural painting object of this study.

This underground building consists of a single-apse rectangular hall oriented along the north–south axis; it is preceded by an endonarthex and surmounted by barrel vaults divided by three arches into four bays and a semi-circular apse [2]. The area of the presbytery of the church preserves very valuable frescoes which still today present open questions and complex problems for a correct historiographic and stylistic interpretation. In the apse, from what emerged from the most recent research, a theory of saints or apostles is depicted with a female figure in the centre, adorned with a halo and bearing an ointment, which could be identified with a representation of the Virgin (Figure 1A). These last frescoes date back to the 9th–10th centuries and present a more direct derivation from the Byzantine stylistic features, being part of a larger composition, which included in the upper part the figure of Christ Pantocrator in an almond, of which today, unfortunately, no traces remain apart from some fragments. On one side of the presbytery, there is a second devotional altar in correspondence with a fresco depicting the *Virgin* in the iconography of the “Galaktotrophousa”, or better represented in the act of breastfeeding the Child, carrying a pomegranate, while a little to the side another fresco represents, in a rigidly frontal pose, Sant’Antonio Abate. Finally, on the two sides of the apse, the Annunciation is depicted: on the left side there is the archangel Gabriel, wrapped in a light drapery that seems to be moved by the wind, and on the right side the Virgin, depicted with precious ornaments holding a book in the left hand (Figure 1B). From a stylistic point of view, the mural paintings outside the semicircular area of the apse can be dated to the 14th century [4].

Generally, in the Middle Ages, the range of materials used for the realization of wall paintings was extremely rich and testifies to the technical ability of medieval artists, as well as to wider phenomena in the medieval world, including the circulation of knowledge, techniques and materials between Europe and the Mediterranean, Africa and Asia [6,7]. In the case of mural paintings, slaked lime and fillers such as sand or ground marble were used for the arriccio and intonaco layers and the palette is less varied than that used for other media. Usually, inorganic pigments were used, because the alkaline environment of lime would be aggressive toward organic pigments [7,8,9,10].

The characterization of raw materials and techniques can help to better understand cultural heritage, i.e., dating and provenance studies or definition of the correct restoration applications and preventive conservation planning [11]. In the case of the mural paintings of the church of Sotterra, in fact, a systematic archaeometric study of a selected area of the mural paintings found in the presbytery area was envisaged. The main purpose was to provide information on the identification of the pictorial materials used in the different chronological phases and to characterize the technical characteristics related to the different styles proposed by art historians [7,9].

In particular, this study preliminarily presents a first step of the entire project and focused on the fresco depicting the Virgin, which is part of the Annunciation (Figure 1). Thanks to the non-destructive investigations carried out in situ (IR imaging, UV-Induced Visible Fluorescence, and X-ray Fluorescence analyses) the mapping and characterization of the pictorial surfaces were obtained. The subsequent analytical studies on micro-samples using polarized optical microscopy (PLOM) and electron scanning microscopy coupled with Energy-dispersive X-ray spectroscopy (SEM-EDS), provided morphological and chemical information on each pictorial layer of the selected mural.

## 2. Analytical Methods

The area of the mural painting depicting the “Virgin” as part of the “Annunciation” (Figure 1) was investigated using both in situ and laboratory-based investigations in order to identify the pigments and characterize the material used.

In particular, the investigation of painted surfaces by in situ methods has proved to be very effective, minimizing contact with already altered surfaces and limiting material sampling. Four different areas were additionally investigated using portable analytical techniques. 

As far as laboratory analyses are concerned, five micro-fragments of the pictorial surface were sampled from areas with already existing lacunae. It is worth underlying that the sampling procedure was conducted according to principles of minimal invasiveness, and taking into account that the wall painting in question was in an advanced state of decay.

**Figure 2:**
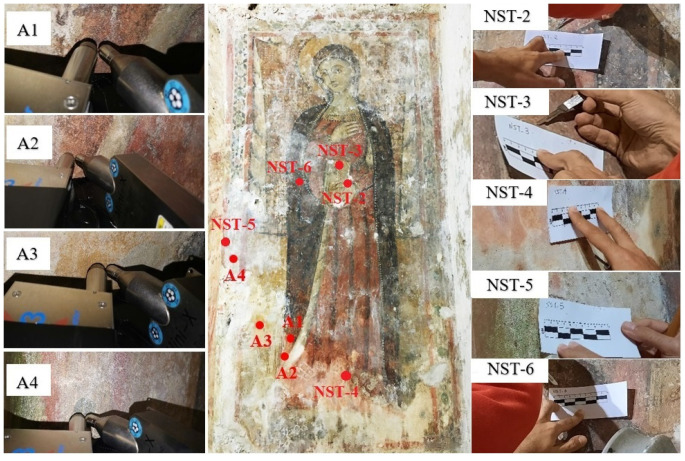
Sampling points for the Mural painting depicting the “Virgin” by in situ methods (ID: A) and laboratory-based analysis (ID: NST).

Details on the sampling areas and micro-fragments are in Table 1 and Figure 2, while all the analytical techniques employed are described below.

### 2.1. In Situ Methods

UV Fluorescence Imaging (UVF Imaging), Infrared Imaging (IR) and portable-X-ray Fluorescence (p-XRF) analysis were carried out inside the Sotterra church by portable equipment.

UVF Imaging is a fundamental technique for documenting the conservation status of the pictorial surface. It allows to study the painting surface and evaluate its state of conservation, helping to identify the inhomogeneities of the surface, as well as to reveal faded or superimposed substances [12]. In particular, this analysis provides a preliminary qualitative mapping of different materials present on the object’s surfaces distinguishing original surfaces from restorations [12,13].

Infrared (IR) imaging is a well-known and non-invasive technique based on wideband imaging in the near-infrared (NIR) range widely used for pictorial surfaces, allowing by looking beneath the visible layers of paint [14,15]. In the case of frescoes, the technique makes it possible to identify the details executed according to “a secco” technique. It is also possible to reveal hidden pictorial layers and to highlight undocumented previous restoration interventions and/or remakes that could not be identified with the naked eye or under UV due to the high fluorescence of the superficial layers (varnish, salts efflorescence, etc). 

The UV and IR multispectral images were acquired using a digital camera with CCD photographic sensor—MADATEC 28.2 MP multispectral system. The UV-induced visible fluorescence acquisitions were performed with UV-IR cut filter (HOYA UV&IR Cut able to cut both UV below 390 nm and IR above 700 nm radiation). Visible fluorescence was induced by two filtered LED sources with emission peak centred at 365 nm, placed at 45 degrees with respect to the observed surface. The IR images were acquired with filters centred at 950 nm, homogeneously illuminating the investigated surface using halogen lamps.

Finally, a non-destructive identification of chemical elements by portable X-ray fluorescence (p-XRF) provided useful data for a preliminary identification of inorganic pigments [16,17,18]. The potential of this elemental analysis technique is amplified if the pictorial surface in which the measurement is performed is well documented. The choice of areas to be analysed could be carried out on the basis of the information provided by UVF and IR imaging [19]. The p-XRF spectrometer used for the multi-elemental analysis of the pigments of the “Virgin” mural painting consisted of an X-ray tube (Mini-X-Amptek) equipped with a Rhodium (Rh) target and operating at a maximum working voltage of 40 kV and maximum current of 0.2 mA. The detector was a silicon Drift Detector system (X-123 SDD—Amptek) with 125–140 eV FWHM at 5.9 keV Mn K line Energy resolution (Depending on peaking time and temperature), collimator 1 mm. The detection range of energy was from 1 keV to 40 keV, with a maximum rate of counts up to 5.6 × 10^5^ cps. The primary beam was positioned perpendicular to the sample, while the detector was positioned at 40 degrees with respect to the primary beam. For the analysis of the selected areas, the following measurement parameters were set in order to ensure a good spectral signal and to optimize the signal-to-noise ratio (SNR): voltage 35 kV, current 80 µA, acquisition time 40 s per area, and working distance 1 cm. The analysed areas are shown in Table 1 and Figure 2.

### 2.2. Laboratory-Based Methods

For laboratory-based analyses such as microscopic investigations by polarized light optical microscopy (PLOM) and SEM-EDS, minimal sampling was needed. Then, a careful sampling of micro-fragments was conducted selecting areas and collecting representative samples for green, yellow, red and black paint. Micro-fragments having sizes smaller than ~2.5 mm^2^ were collected from the painted areas following minimally invasive procedures, by using suitable stainless-steel tools such as small tweezers, scalpels and micro-scalpels. In particular, micro-fragments of the pigmented surface were sampled close to the already existing lacunae and damaged portions.

Specifically, polarized light optical microscopy (PLOM) on thin stratigraphic sections was conducted in order to characterize materials used in the mural painting as well as pigmenting compounds. Observations were performed using a Primotech 40 (Primotech Zeiss) microscope coupled with a digital camera to capture images. Observations were carried out on NST-3, NST-4 and NST-5 samples. On the contrary, thin sections could not be made for the NST-2 and NST-6 samples due to their extreme fragility and thinness. 

SEM-EDS was performed on the painted samples surfaces to obtain information about morphology and chemical composition (in terms of major elements) [11,19,20,21] by Ultra-High-Resolution SEM (UHR-SEM)—ZEISS CrossBeam 350 equipment, coupled with a spectrometer EDS—EDAX OCTANE Elite Plus—Silicon drift type. Investigations were performed on samples coated with a thin and highly conductive graphite film, both on thin sections (i.e., NST-3, NST-4 and NST-5) and on very small pigmented fragments (NST-2, NST-6). Instrumental conditions set for EDS analysis were HV: 15 keV and probe current: 100 pA.

## 3. Results

### 3.1. In Situ Methods

**Figure 3:**
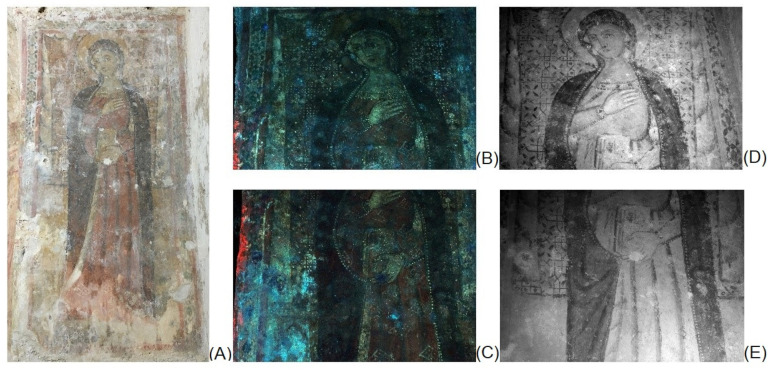
Virgin mural painting: Photographs by visible light (**A**), UV fluorescence images (**B**,**C**), Infrared images (950 nm) (**D**,**E**).

**Figure 4:**
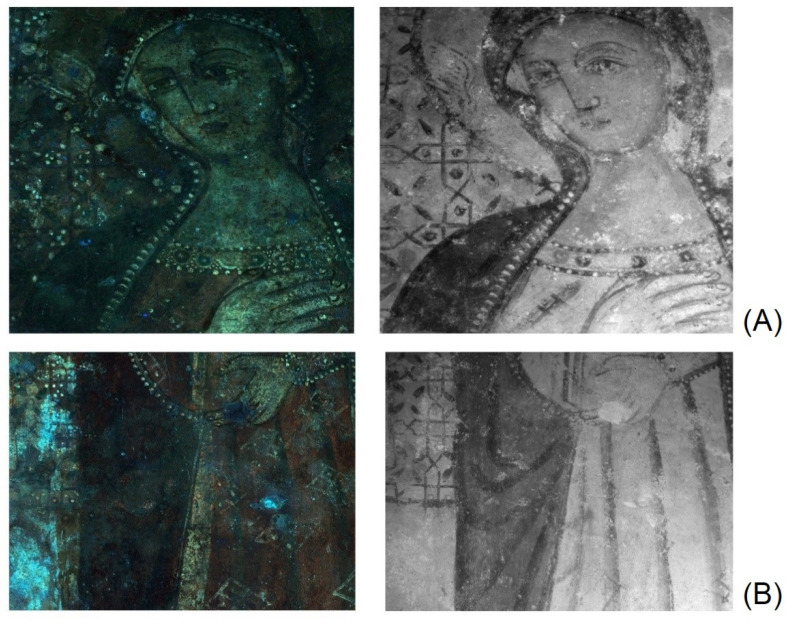
Details (at different magnifications) of the (from left) UVF and IR images for the comparison of the diagnostic information obtained from the imaging techniques: (**A**) Detail of the face and the halo; (**B**) detail of the dress and of the background floral-geometric decoration.

UVF images show a brilliant red luminescence emission on the right edge attributable to a biological growth (Figure 3A–C). The blue-whitish fluorescence colour localizes the patinas present on the calcareous encrustations and efflorescence salts on the pictorial surface. Moreover, the contours of the lacunae of pictorial layers are highlighted and documented (Figure 3B,E and Figure 4).

Thanks to the IR transparency of the surface whitish patinas (efflorescence salts) and to the infrared absorption of the pigments, the IR images allowed to obtain a better reading of the whole representation (Figure 3A,D,E). The details of the face, dress and mantle of the Virgin are characterized by a more complex study of shadows, not appreciable to the naked eye due to its poor conservation state (Figure 4). The halo on the left side of the observer shows an iconographic detail almost imperceptible to the photographic documentation both in IR and in UV radiation (Figure 4A,B): it is probably a dove that could be the subject of future investigations for historical and artistic studies of the style of this portion of the cycle pictorial.

The IR images also provide a clear documentation of the incisions made to trace the underdrawing of the geometric decorations in the background as well as the curtains with Cosmatesque style decoration. In particular, the side drapes recover their original depth thanks to the better reading of the use of “chiaroscuro” for the rendering of the volume (Figure 33D and Figure 4B). Moreover, IR image localises the “a secco” details (transparent to IR) such as the small white circular shapes on the background floral-geometric decoration (Figure 4). The IR documentation of these recovered figurative details hardly perceptible to the naked eye can support the future cleaning operations.

**Figure 5:**
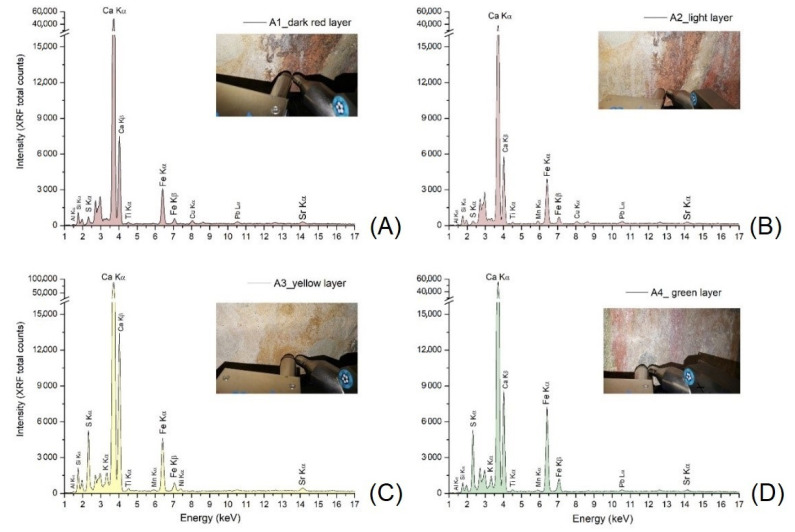
p-XRF spectra acquired on the “Virgin” mural painting; A1, Dark Red layer (**A**); A2, Light Red layer (**B**); A3, Yellow layer (**C**); A4, Green layer (**D**).

Starting from the information obtained from the macroscopic observation of the pictorial surfaces and from the diagnostic UV and IR imaging, XRF was carried out on 4 sample areas for the identification of the pigments used (Table 2 and Figure 5).

The presence of pigments based on iron and other elements typical of clay minerals has been verified for the red, yellow and green pictorial layers which seems to be made of red, yellow and green earths, respectively. [22,23,24,25,26,27,28,29]. The different shades of red colours were obtained from a different mixing and/or overlapping of the red and yellow (background) pigments, as suggested by Mn in A2–A4 spectra (Figure 5). Moreover, the copper (Cu) traces detected in A1 and A2 XRF spectra are likely due to impurities of clays present in the composition of red earthy pigments.

Yellow and green (A3 and A4 spectra in Figure 5, respectively) are characterized by a higher content of sulphur probably due to the degradation process of plaster layers [9,10].

The inorganic chromophores identified by non-destructive investigation are typical of the fresco techniques from ancient times and they do not represent a specific marker of a local production or manufacturing period.

From a mineralogical point of view, red and yellow earths are natural mixtures composed clay minerals that can form the predominant part of the material, coloured with iron oxides or also Mn oxides [22,23,26,27,28,29]. Finally, green earth (where celadonite, K [(Al, Fe^3+^), (Fe^2+^, Mg)] (AlSi_3_, Si_4_) O_10_(OH)_2_ and glauconite, (K, Na) (Fe^3+^, Al, Mg)_2_(Si,Al)_4_O_10_(OH)_2_ constitute its main colouring agents) includes siliceous minerals of dull greyish green colour widely used by the artists on different pictorial techniques [29,30,31,32]. 

### 3.2. Laboratory-Based Methods

As PLOM observations are concerned, the samples were described according to the main mineralogical-petrographic features and also considering the presence of overlapping layers. In particular, they have been characterized on the basis of the type/sorting of the aggregate and on the main features of the binder present in each layer.

**Figure 6:**
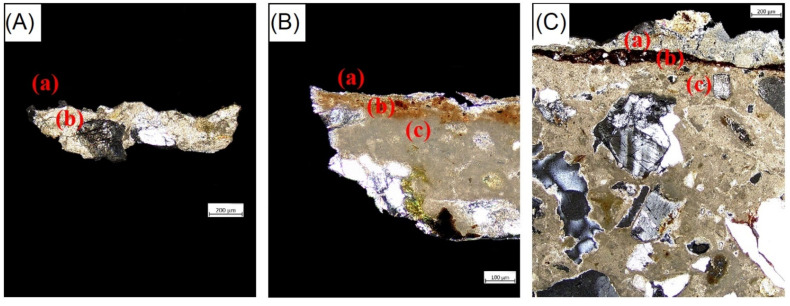
Photomicrographs by polarized light optical microscopy; Sample NST-3 (5×, CPL, scale-bar 200 µm) (**A**), Sample NST-4 (10×, CPL, scale-bar 100 µm) (**B**) and Sample NST-5 (5×, CPL, scale-bar 200 µm) (**C**). Note: CPL = Crossed Polarized Light.

All samples (Figure 6A–C) consist of a plaster layer (c) having a fairly homogeneous and compact micritic binder. This layer is particularly evident in NST-4 and NST-5 while it is not very representative for the NST-3 sample as the sample is too small. The aggregate fraction is moderately sorted and mostly constituted by monocrystalline quartz granules and polycrystalline ones, followed by calcite, feldspars, micas and iron oxides with sizes up to 600 μm, varying in shape from sub-rounded to sub-angular. The percentage of the aggregate is about 35% (area) [33]. The latter was assessed by means of a semi-quantitative visual estimation. The porosity varies from about 10 to 15% (area) and it is made up of both primary and secondary pores with sizes up to 400 μm. In addition, recrystallization phenomena of calcite are observed in the voids [34].

Above the lower plaster layers, the three samples show a thin pigmented layer, still appreciable, especially in NST-4 and NST-5. This layer has a thickness of 80 μm in NST-4 sample [layer (b)], up to 100 μm in NST-5 [layer (b)], while in NST-3 it is poorly distinguishable with dimensions less than 10 μm [layer (a)]. Within the pictorial layers, iron oxides followed by a few small crystals of quartz and micas were detected.

In samples NST-4 and NST-5, a third layer (a) above the pigmented one was recognized, with thicknesses ranging from 10 to 200 μm. It is plaster, with an inhomogeneous cryptocrystalline binder, showing microfractures and rare inclusions of tiny quartz and micas.

**Figure 7:**
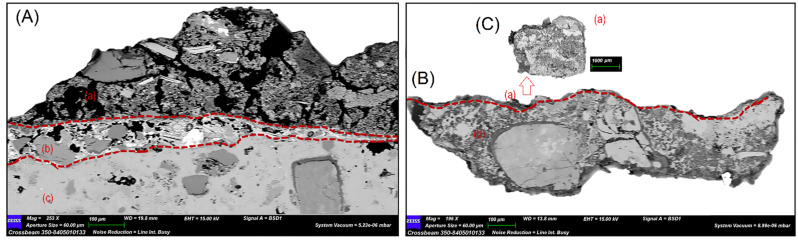
SEM representative images, showing samples’ stratigraphy on thin sections. Specifically: (**A**) sample NST-5 with evidence of 3 layers named as (**a**–**c**); (**B**) sample NST-3 with evidence of 2 layers named as (**a**,**b**). All layers were analysed by EDS (Table 3); for NST-3 analysis was also performed on a residual of micro-fragment (**C**) related to layer (**a**) of the same samples because the pigmented layer had clearly detached from the stone support during section preparation.

SEM-EDS analyses were performed both on micro fragments and on thin sections (Table 3 and Figure 7), investigating the pigmented layers and that underneath, when present. In the case of NST-2 and NST-6, as specified above, it was not possible to make thin sections, therefore investigations were carried out directly on the micro-fragments.

Moreover, the study of sample NST-3 was performed both on micro-fragment and thin section, because the pigmented layer had clearly detached from the support during the preparation of the section, making the pictorial layer very thin and difficult to analyse. Table 3 provides a summary of the data, where for each sample the main elemental composition of paint layers, overlying and/or underlying ones is reported.

## 4. Discussion

A preliminary macrophotography and raking light documentation allowed to distinguish the different pictorial layers used in the “Virgin” mural painting at Sotterra church highlighting the use of a typical palette of yellow, green, light red, dark-red and black pigments [30]. Moreover, the conservation state (lacunae, decohesion, salt efflorescence) of this mural painting has been revealed. The images obtained by UVF imaging show the presence of biological growth characterized by a red fluorescence response, typical of biological activity [12]. This phenomenon is typically made easy due to the microclimatic condition that occurs in an underground site [35,36] (temperature 20.7 °C and relative humidity 82% values measured at moment of in situ diagnostic investigation). Infrared imaging revealed interesting technical and iconographic details of the representation. Moreover, the complementary way UVF and IR imaging were used represent a useful tool to obtain information on details that are not visible to the naked eye. The refined use of *chiaroscuro* to create the volumes of the figure against the background clearly emerged. No variations were observed with respect to the initial composition and a first trace of the outlines observed, retraced in the final phase with a black line along the outer edges of the figure. In the initial design phase, the geometric decoration of the background is performed by engraving on the still wet plaster.

Finally, the UV fluorescence and IR images highlighted the presence of an iconographic detail not directly perceptible to the naked eye: it is probably a small dove on the Virgin halo. The improved legibility of the figure of the Madonna as well as of the Cosmatesque decoration on the drape in the background allows comparative studies with other contemporary examples [37,38] for the best definition of the period and of the artists who worked on the most recent phase of the Sotterra pictorial cycle.

The main colours of the representation (red in different shades, yellow and green) were analysed by p-XRF in a non-invasive way for a preliminary and rapid assessment of different chromophores and to guide the choice of sampling points [39,40,41]. Calcium (Ca) was found in all areas analysed as a chemical element present with the highest XRF signal counts. This element is certainly attributable to the original plaster but also to the traces of surface plaster due to previous restorations as demonstrated later by the observation of thin sections. The presence of strontium (Sr) and sulphur (S) was also systematically found: the first is a typical impurity in Ca-compounds (sulphates and carbonates) for its geochemical affinity with Ca while S can be attributed to the composition of degradation products (due to the sulfation of the original layers). The major elements attributable to the composition of the pigments are iron (Fe), silicon (Si), aluminium (Al) with traces of titanium (Ti) and manganese (Mn), a typical composition of pigments based on iron oxides and/or clay minerals (red, yellow and green earthy pigments) [22,23,27,28,29,30,31,32]. Potassium (K) was revealed only in green and yellow pictorial layers. Traces of lead (Pb) were also revealed in the XRF spectra. This chemical element is to be attributed to restoration products (surface plaster or pictorial retouches) and not to the original plaster as demonstrated by the SEM-EDS analysis performed on thin sections.

Microscopic observations under PLOM allowed distinguishing different layers among samples. Generally, for all the samples, the lower layers in contact with the masonry, are characterized by a lime-based binder, rich in oxides, silicate and clayey minerals. Moreover, within the pictorial layers, iron oxides associated with crystals of quartz and micas were detected in all the samples. Only in NST-4 and NST-5 a third level was detected, poorly homogeneous and heavily fractured with cryptocrystalline binder and few accessory mineralogical phases such as quartz and micas.

As a consequence of the presence of such layer superimposed on the pigmented one for both, it is reasonable to assume that the wall paint has undergone restoration works/remaking. Compared to the original plaster, the restoration layer is less homogeneous and more porous, with the presence of micro-cracks; mineralogically, no feldspar and Fe-oxides are detected. This layer was applied on portions at risk of falling as a protective support to avoid the loss of the painting that was in poor conservation conditions. However, there is no written evidence of this.

As far as samples stratigraphy is concerned, SEM observations carried out on thin sections confirm results obtained by PLOM. As for the sample coming from the golden decorations of the Virgin’s dress, consisting of two layers, chemical investigations suggest the presence of a lime-based plaster, the possible use of raw materials from clay and other minerals common in soils, surely used as aggregate [23,30,31,39,40]. As regards the yellow-coloured layer, the EDS investigations were conducted on a residue of painted micro-fragment showed that the composition of this layer seems very similar to the underlying layer (i.e., Ca, Si, Mg, Al, Fe) differing only from the presence of S and traces of Na and P. Even in this case, data suggest the use of earthy pigments for the yellow tones.

Natural iron oxide and/or clays-based mixture of pigments have been widely employed for artistic purposes since prehistoric times thanks to their availability, stability, and the wide range of colours/hues. In particular, red, green, yellow and brown earthy pigments were commonly used for their perfect compatibility for the fresco technique [39,41]. The great stability of these mineral pigments used in the fresco technique is also evident in the mural paintings of the Sotterra church. In fact, despite their long state of abandonment and oblivion and the difficult conservation conditions due to the hypogeum environment, a sufficiently good conservation of the pictorial layers and adhesion to the substrate were observed.

Going on for the elemental composition, is mainly based of Ca, Mg, Al, S and Fe, with traces of Na, Cl, K, P and Cl. These elements allowed to hypothesize the use of natural earthy pigments as chromophore substances for the dark colour, for example, made of iron oxides, mixed with other minerals common in soils. Furthermore, given the P content, the use of a pigment of organic origin, such as bone black, probably mixed to earths to darken the brownish tones, cannot be excluded as well as the use of an organic protective compound. It is possible that P and Cl detected in the more superficial layers have an environmental origin. Finally, the presence of Ca in both micro-fragments is almost surely attributable to the preparatory layer.

## 5. Conclusions

One of the most important objectives in the field of heritage science is to acquire comprehensive knowledge to define suitable conservation and restoration procedures.

Therefore, correct conservation and restoration works require a better knowledge of the raw materials, of the degradation phenomena and of the past conservative treatments. In this way, information about the material and the techniques used by the artists should be carefully investigated following a multi-analytical approach.

In this work, a combined multi-analytical approach, involving both in situ and laboratory-based methodologies, was successfully applied on what is considered as one of the mural Calabrian masterpieces inside the Sotterra church in Paola (Calabria region, Italy). Diagnostic investigations revealed the raw materials and pictorial technique used for one of the wall paintings of the valuable cycle of the Sotterra church, have so far never been analysed from a technical-scientific point of view.

In particular, the non-destructive investigations by IR imaging, and UV- Induced Visible Fluorescence provided mapping and characterization of pictorial layers and the first data about deterioration phenomena and the executive techniques. Then, the employment of portable X-ray fluorescence (p-XRF) spectrometry and scanning electron microscopy (SEM) coupled with energy-dispersive spectroscopy (EDS), allowed to characterize at the elemental scales, the composition of the raw materials used in the mural painting both in terms of preparatory and pigmenting agents, as well as their stratigraphy and morphology. The latter was further investigated by optical microscopy (PLOM) with an evaluation of the mineral-petrographic properties.

The results provided useful data for a better knowledge of the mural painting technique and were also crucial in establishing the state of conservation of the painting as well as whether possible scarcely or non-documented restoration interventions took place. 

Furthermore, the presence of similar elements/compounds for all the examined samples allowed to attest to the use of a lime-based plaster rich in silicate minerals and iron oxides for the preparatory layers. In the painted layers, on the other hand, the use of inorganic chromophores based on natural mineral pigments prevails for the red, green and yellow shades, probably mixed with the use of organic compounds as in the case of the blackish micro-fragment. Finally, in the more superficial layers of some samples, a third layer was identified, consisting of a lime-based plaster different from the preparatory layer. From a compositional point of view, this layer clearly suggests that the mural paintings have undergone renovations/remaking over time, of which there is no clear scientific documentation to date.

The main goal of this multimethodological approach was to determine the chemical composition of the frescoes, discriminate pigments and raw materials, and provide suitable information to restorers for planning future restoration work. In fact, in the context of scientific research performed on artworks, this approach represents a necessary prerequisite for the adoption of the best cleaning and protection strategies. Further studies will be devoted to a deep comprehension of alteration and degradation phenomena.

## Figures and Tables

**Table 1:** Mural painting depicting the “Virgin”: an overview of areas investigated and sampled both with in situ and laboratory-based methods.

*Area ID*	*Measurement Area*	*Color*	*Employed Techniques*
A1	Mantle of Virgin	Dark Red layer	p-XRF
A2	Mantle of Virgin	Light Red layer	p-XRF
A3	Background	Yellow layer	p-XRF
A4	Detail of geometric decoration of the frame	Green layer	p-XRF
***Sample ID***	***Sampling Area***	***Color***	***Employed Techniques***
NST-2	From the bracelet of Virgin	Green	SEM-EDS
NST-3	From the book that is in the Virgin’s hand	Yellow	PLOM, SEM-EDS
NST-4	From the bottom part of the Virgin’s mantle	Reddish/whitish	PLOM, SEM-EDS
NST-5	From the left border of the mural painting	Reddish/whitish	PLOM, SEM-EDS
NST-6	From the left part of the Virgin’s mantle	Blackish	SEM-EDS

**Table 2:** Portable X-ray fluorescence (p-XRF) elemental composition of “Virgin” mural painting. Notes: more abundant elements (in bold); minor or trace elements (in brackets).

*Area*	*Type*	*Chemical Elements by XRF*	*Identified Pigment*
**A1**	Dark red layer	**Ca, Fe, S, Si, Al** (Ti, Cu, Pb, Sr)	**Red Earth**
**A2**	Light red layer	**Ca, Fe, S, Si, Al** (Ti, Mn, Cu, Pb, Sr)	**Red Earth**
**A3**	Yellow layer	**Ca, S, Fe, Si, K, Al** (Ti, Mn, Ni, Sr)	**Yellow Earth**
**A4**	Green layer	**Ca, Fe, S, K, Si, Al** (Ti, Mn, Pb, Sr)	**Green Earth**

**Table 3:** EDS chemical data detected for both thin sections and micro-fragments of “Virgin” mural painting. Notes: more abundant elements (in bold); minor elements (in brackets); / (not detected); * the pigmented surface layer of NST-3 was analysed on a micro-fragment.

*Samples ID*	*Layers*	*Type*	*Chemical Elements by EDS on Thin Sections*
**NST-3**	**Layer** (**a**) *	**Layer with yellow pigment**	/
	**Layer** (**b**)	**plaster**	**Ca, Si, Mg, Al, Fe** (K)
**NST-4**	**Layer** (**a**)	**plaster**	**Ca, Si, Mg, Al, Fe** (Sr, S, Na, K, Pb, Cl)
	**Layer** (**b**)	**Layer with red pigment**	**Ca, Si, Fe, Mg, Al** (Sr, S, Na)
	**Layer** (**c**)	**plaster**	**Ca, Mg, Si, Fe** (Al, S)
**NST-5**	**Layer** (**a**)	**plaster**	**Ca, Fe, Si, Al, Mg, K** (Ti, Ba, S, Cl)
	**Layer** (**b**)	**Layer with red pigment**	**Ca, Si, Mg** (Na, Al, S, Fe, Cl)
	**Layer** (**c**)	**plaster**	**Ca, Mg, Si, Fe** (Al, S, Na)
***Samples***	***Areas***	***Type***	***Chemical Elements by EDS on Micro-Fragments***
**NST-2**	**Surface**	**Green micro-fragment**	**Ca, Si, Mg, Al, S** (Na, P, Cl, K, Fe)
**NST-3**	**Surface**	**Yellow micro-fragment**	**Ca, Si, Mg, Al, Fe** (K, Cl)
**NST-6**	**Surface**	**Blackish micro-fragment**	**Si, Ca, Mg, Al, S, Fe** (Na, Cl, K, P)
